# Surface Alloying in Silver-Cobalt through a Second Wave Solution Combustion Synthesis Technique

**DOI:** 10.3390/nano8080604

**Published:** 2018-08-09

**Authors:** Anchu Ashok, Anand Kumar, Faris Tarlochan

**Affiliations:** 1 Department of Mechanical and Industrial Engineering, College of Engineering, Qatar University, Doha P.O. Box-2713, Qatar; anchuashok@qu.edu.qa (A.A.); faris.tarlochan@qu.edu.qa (F.T.); 2 Department of Chemical Engineering, College of Engineering, Qatar University, Doha P.O. Box-2713, Qatar

**Keywords:** bimetals, solution combustion synthesis, second wave combustion synthesis, Ag-Co nanoparticles

## Abstract

Herein, we report the synthesis of silver-cobalt nanopowders using three different modes of solution combustion synthesis, and we present the effects of the synthesis conditions on particle morphology. The synthesized nanoparticles were characterized using X-ray diffraction (XRD), Scanning electron microscopy (SEM), Fourier transform infrared spectroscopy (FTIR), UV-Visible spectrophotometer (UV-vis), Transmission electron microscopy (TEM), and X-Ray Photoelectron Spectroscopy (XPS) to understand the structural and elemental properties. When Co is synthesized over Ag in a second wave of combustion, peak shifts observed in XRD and XPS show a change in the cell parameters and prove the existence of a strong electronic interaction between Ag and Co. Better control of mixing and alloying through the second wave combustion synthesis mode (SWCS) was evident. The sequence of combustion affects the structure and composition of the material. SWCS reduces the amount of carbon content, as compared to single-stage combustion, and the combustion of carbon is followed by a rearrangement of atoms.

## 1. Introduction

Bimetallic nanoparticles have received great attention due to their multifunctionalities, selectivity, and activity over monometallic particles, and they are increasingly being applied to various areas, including electronics, optics, catalysis, and magnetics [1,2,3,4,5,6]. Bimetallic nanoparticles with different structures, like crown-jewel, hollow, heterostructure, core-shell, alloys, and porous structures, have been synthesized using various techniques, such as chemical methods, hydrothermal, impregnation, sol-gel, spray pyrolysis, and precipitation methods [7,8,9,10,11]. This structural diversity is due to the atomic distribution of the individual metals in bimetals. The chemical and physical properties of bimetals are derived from the synergetic factors of the two constituent metals [12,13,14].

Bimetallic silver-cobalt (Ag-Co) has gained interest in different branches of science and industry, including catalysis [15,16], biotechnologies [17], and energy storage and conversion devices such as fuel cells, batteries, etc. [18,19,20]. Different techniques have been used for the preparation of bimetallic Ag-Co, including hydrothermal reduction, sol-gel, intermatrix synthesis, co-precipitation, and so on [15,17,21]. Erdogan et al. synthesized bimetallic Ag-Co using a co-precipitation method and studied the catalytic oxidation of carbon monoxide [21]. Alonso and co-workers tested the bacterial disinfection property of fibrous polymer Ag/Co composites and compared their bactericidal activity with Ag and Co monometals [17]. Lima et al. designed bimetallic silver-cobalt particles with different molar ratios for oxygen reduction in an alkaline medium [20]. In this work, we used solution combustion synthesis for nanoparticle preparation and studied the effect of synthesis conditions on the surface and bulk structures.

Solution combustion synthesis (SCS) is a commonly used simple and cost-effective synthesis approach for the preparation of nanomaterials with high surface areas and crystallinity. SCS involves a self-sustained exothermic reaction in a homogeneous solution of metal nitrate as an oxidizer and fuel as a reducer (e.g., glycine, urea, glucose, hydrazine, etc.). The energy needed for the high-temperature reaction is obtained through the decomposition of the metal nitrate and added fuel. The reactive groups—ammonia (NH_3_) in fuel, and nitric acid (HNO_3_) from the metal nitrate—forms a reactive atmosphere that triggers the combustion. This highly exothermic reaction provides the energy to form nanoparticles with high purity and crystallinity without any post-synthesis treatments (e.g., purification and calcination) [22,23,24,25,26,27]. The stoichiometric combustion reaction between an oxidizer and glycine can be represented by the following equation:

(1) $$M^{V}{\left({\mathrm{NO}}_{3}\right)}_{V}+\left(\frac{10}{9}\varphi \right){\mathrm{NH}}_{2}{\mathrm{CH}}_{2}\mathrm{COOH}+\frac{10}{4}\left(\varphi -1\right)O_{2}\;M^{V}O_{\frac{V}{2}}\left(s\right)+\left(\frac{20}{9}\varphi \right){\mathrm{CO}}_{2}\left(g\right)+\frac{25}{18}\varphi H_{2}O\left(g\right)+\left(\frac{5\varphi +9}{9}\right)N_{2}\left(g\right)$$

where $M^{V}$ is a metal with *ν* valency, and *φ* is the fuel to oxidizer ratio; *φ* = 1 is the stoichiometric condition where solution does not require atmospheric oxygen for complete combustion, *φ* > 1 is a fuel-rich condition, and *φ* < 1 is a fuel-lean condition. The *φ* value has a significant role in controlling the phase, morphology, and composition of the nanomaterials. Using SCS, it is possible to synthesize a variety of metals, metal-oxides, alloys, and ceramics with specific properties and features [28,29,30]. Herein, we report the enhancement of alloying bimetallic Ag-Co using second wave solution combustion synthesis (SWSC).

## 2. Experimental Section

**Scheme 1:**
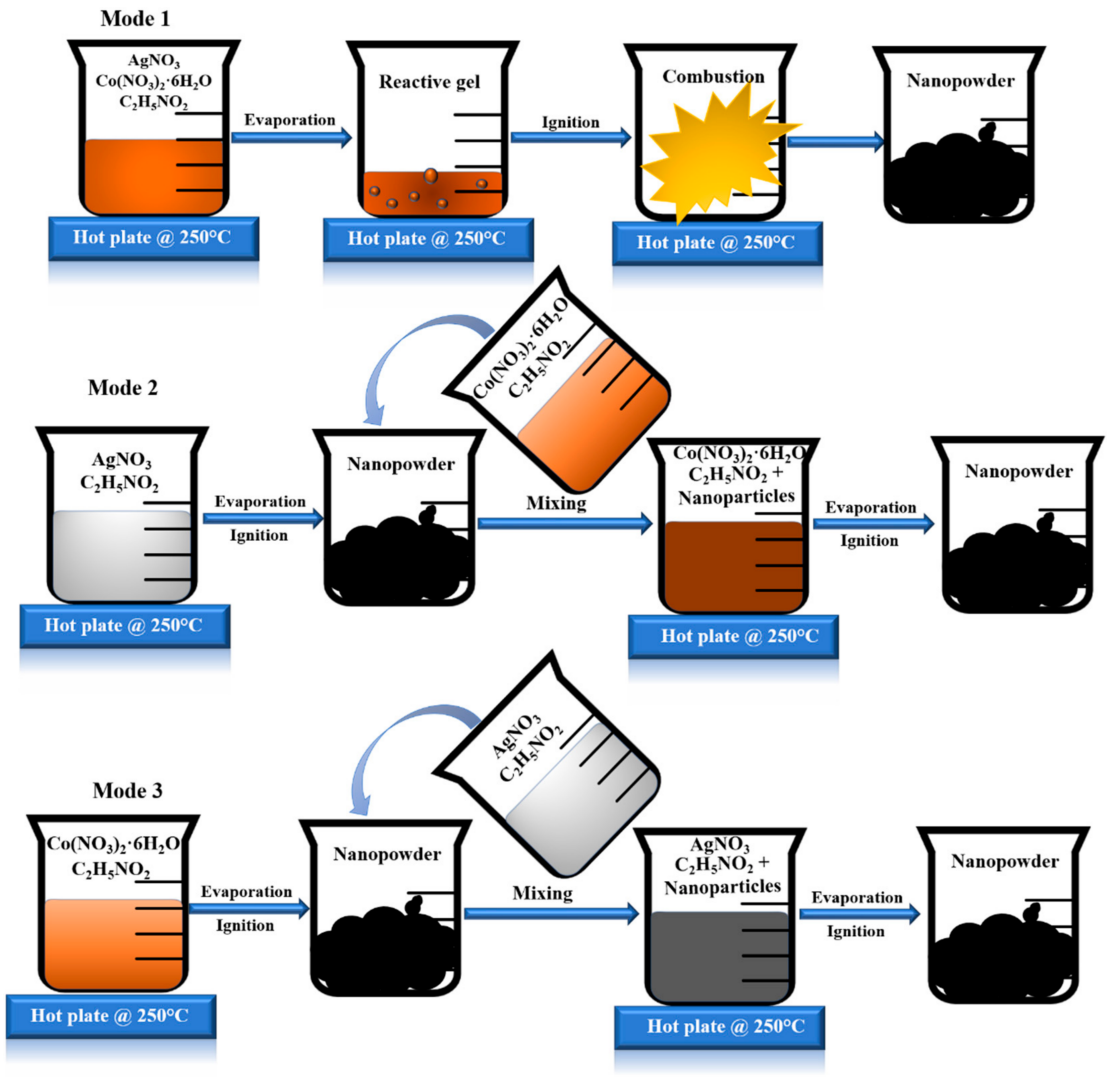
Schematic representation of three different modes of solution combustion synthesis.

Bimetallic silver-cobalt was synthesized using three different modes of solution combustion synthesis with a 1:1 weight ratio. The synthesis process is schematically represented in Scheme 1. It should be noted that the three synthesis modes are termed AgCo-11, AgCo-12, and AgCo-21 for the sake of convenience, and they do not necessarily represent the metallic forms of Ag and Co. As will be clear in subsequent sections, in most cases, cobalt is present in an oxidized state.

### 2.1. Mode 1: AgCo-11

A homogeneous aqueous solution of silver nitrate (AgNO_3_), cobalt nitrate Co(NO_3_)_2_·6H_2_O, and glycine (C_2_H_5_NO_2_) with a fuel to oxidizer ratio (*φ*) of 1.75 was prepared by mixing the desired molar ratio of the precursors in 25 mL of water. The quantity of the precursors was measured based on the preparation of 1.5 g of products using stoichiometric values, as shown in Equation (1). The dissolved precursors were heated over a hot plate at 250 °C until the water evaporated, and the reactive gel reached its auto-ignition temperature. Thereafter, the self-sustained combustion wave propagated from one end to the other end of the beaker by converting the precursors to metal/oxide nanopowders.

### 2.2. Mode 2: AgCo-12

During the first step, the silver nitrate (AgNO_3_) and glycine (C_2_H_5_NO_2_), with fuel to oxidizer ratio (*φ*) of 1.75, were mixed together in 25 mL of deionized water and combusted in a single step, as shown in Mode 2 (Scheme 1), to produce silver nanoparticles. The particles thus formed were crushed and sieved to obtain uniformly sized particles. In the second step, a solution of cobalt nitrate (Co(NO_3_)_2_∙6H_2_O) and glycine (C_2_H_5_NO_2_) was mixed with the silver nanoparticles synthesized in the first step. This solution was mixed thoroughly by dispersing the nanopowders completely, thereupon the resulting mixture was combusted over the hot plate heater. This combustion mode is termed the second wave combustion synthesis (SWCS), and it usually involves two stages consisting of solution combustion synthesis. In this mode, the cobalt/cobalt oxide particles are expected to be on the top of the silver nanoparticles that were synthesized in the first stage of combustion.

### 2.3. Mode 3: AgCo-21

In mode 3, a cobalt nitrate (Co(NO_3_)_2_∙6H_2_O) and glycine (C_2_H_5_NO_2_) solution was combusted to synthesize cobalt/cobalt oxide nanoparticles initially. These nanopowders are mixed with silver nitrate (AgNO_3_) and glycine (C_2_H_5_NO_2_) solution followed by second wave combustion. Here, silver particles are expected to be on the surface, possibly covering the cobalt nanoparticles. The obtained nanopowders from the three modes were crushed using a hand motor and sieved to obtain particles <75 µm. These particles were characterized using different techniques to identify the structure and composition of the synthesized nanopowders.

## 3. Material Characterization

A software package “THERMO” was used to conduct the thermodynamic analysis of the combustion synthesis under adiabatic condition. XRD measurements were carried out using MiniFlex II Desktop X-ray powder diffractometer (Rigaku, Leatherhead, UK) with Cu-Kα radiation with a 28–80° scan range to identify the crystallinity of bimetallic Ag-Co. A Thermo Fisher Scientific Evolution 300 UV-Visible spectrophotometer (Waltham, MA, USA) was used to identify the optical properties of the synthesized samples. A measured amount of sample was dispersed in water using an ultrasonic device (iSonic, Chicago, IL, USA) to obtain a homogeneous solution that was used for UV-Vis measurements. The chemical bonding on the catalyst surface was studied using Fourier transform infrared spectroscopy (Thermo Fisher Scientific, Nicolet FTIR 6700, Waltham, MA, USA) in the range of 500–900 cm^−1^. SEM (Thermo Fisher Scientific, Nova Nano 450 FEI, Waltham, MA, USA) was used to study the surface morphology of the catalysts. FEI Talos F200X TEM coupled with EDS (FEI Super X EDS system) was used to identify the particle size and for elemental mapping. To prepare the sample for TEM analysis, the synthesized nanoparticles were dispersed in ethanol and sonicated for 30 min. A volume of 20 µL of this solution was dropped over a carbon-covered 200-mesh copper grid and allowed to dry at room temperature before analysis. XPS (Kratos AXIS Ultra DLD, Manchester, UK) was used to identify the bonding configuration and oxidation state of Ag and Co on the surface of the as-prepared samples.

## 4. Results and Discussion

### 4.1. Thermodynamic Analysis

**Scheme 2:**
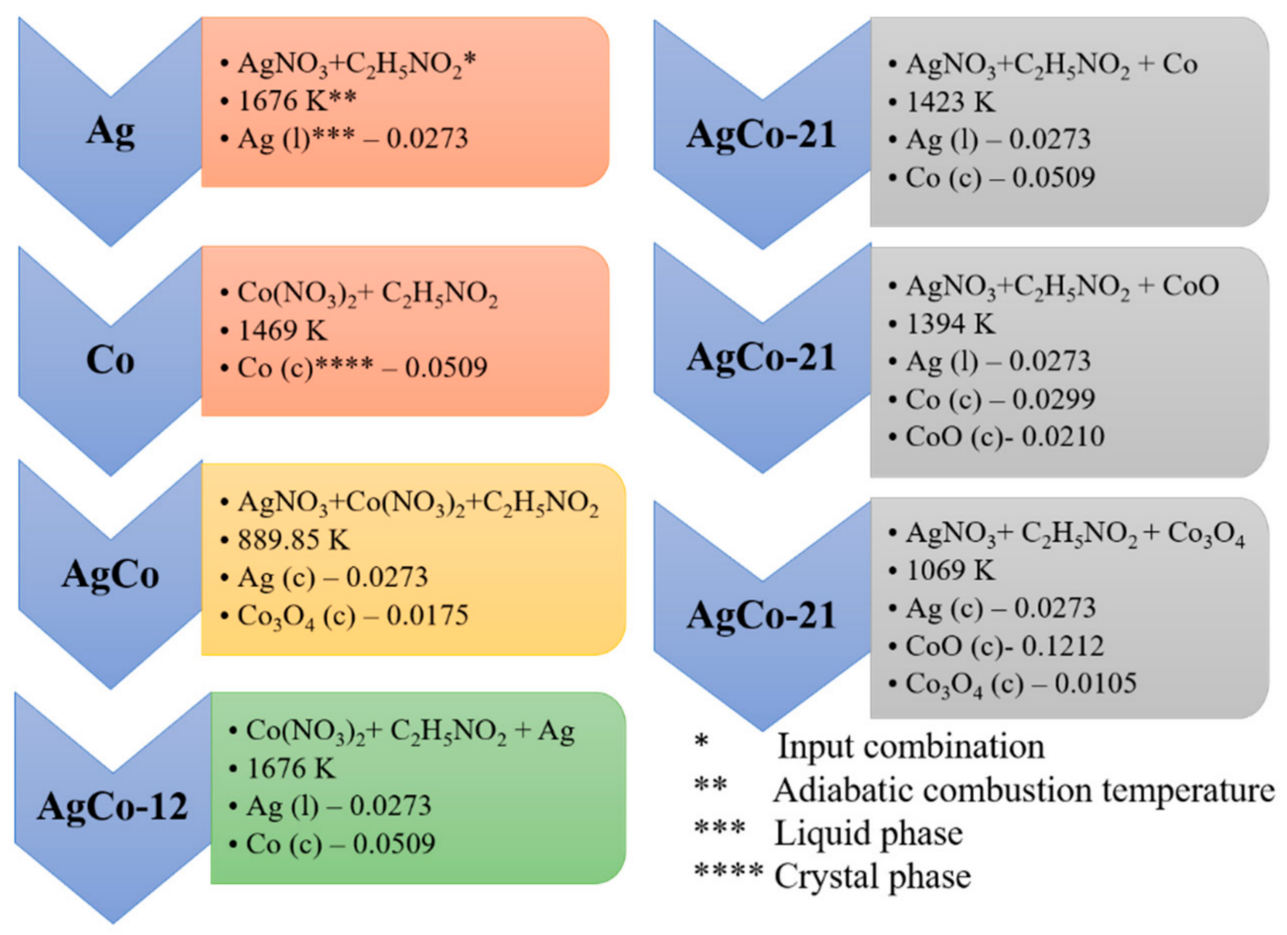
Adiabatic combustion temperatures and the output products in the three modes of combustion.

Detailed thermodynamic analysis of all the expected combustion paths are illustrated in Scheme 2. Silver at *φ* =1.75 produces an adiabatic combustion temperature of 1676 K, resulting in silver in the liquid phase. The melting point of Ag is 1234 K, and any temperature higher than this value causes its conversion from the solid phase to liquid form. The adiabatic combustion temperature of Co is 1469 K and AgCo-11 is 889.85 K. The cobalt nitrate/glycine mixture at *φ* =1.75 yields pure metallic cobalt, and the silver nitrate/cobalt nitrate/glycine (Ag-Co) system furnishes metallic Ag crystals and cobalt in the Co_3_O_4_ phase. In AgCo-12, cobalt nitrate/glycine combustion takes place in the presence of the silver nanoparticles prepared during the first stage of combustion, the whole system reaches an overall combustion temperature of 1424 K, and the output phases at the adiabatic combustion temperature are liquid Ag with solid metallic cobalt. On the other hand, in AgCo-21, there are three possible Co species—Co, CoO, and Co_3_O_4_—for the second phase of combustion. AgCo-21 with Co reaches the combustion temperature of 1423 K, generating a Ag liquid phase and the metallic form of cobalt. When using CoO as the input in second stage, the output product distribution is a mixture of Ag (liquid), CoO, and Co at 1394 K. The partial reduction of CoO to Co was possibly achieved during the combustion of the silver/glycine system due the reducing environment created by the product gases. Similarly, Ag(NO_3_)/C_2_H_5_NO_2_ combustion in the presence of Co_3_O_4_ gives metallic silver at 1069 K, along with the conversion of 88% of Co_3_O_4_ to CoO. During the second wave combustion, there is a possibility of the gaseous mixture creating a reducing environment, which reduces the existing oxides formed during the first stage of combustion.

The sequence of the reaction in bimetallic combustion can be explained in terms of the decomposition temperatures of silver nitrate and cobalt nitrate obtained from the literature, that is, 473 K and 448 K, respectively [31,32]. Once the temperature of the cobalt nitrate/silver nitrate/glycine mixture reaches 448 K, cobalt nitrate starts to decompose, possibly leading to an exothermic combustion reaction that increases the temperature and subsequently starts the combustion of silver/nitrate/glycine. In this sequence, Ag is anticipated to be on the surface of cobalt/cobalt oxide, even though it involves a single-step combustion synthesis. This sequence is similar to the steps involved in AgCo-21, which is accomplished in two stages. The silver nitrate/glycine combustion is performed in the presence of the cobalt oxide that was synthesized earlier. Based on the order in which the combustion reaction takes place in the Ag-Co system, some similarities between the properties of AgCo-11 and AgCo-21 are anticipated.

### 4.2. Experimental Analysis

**Figure 1:**
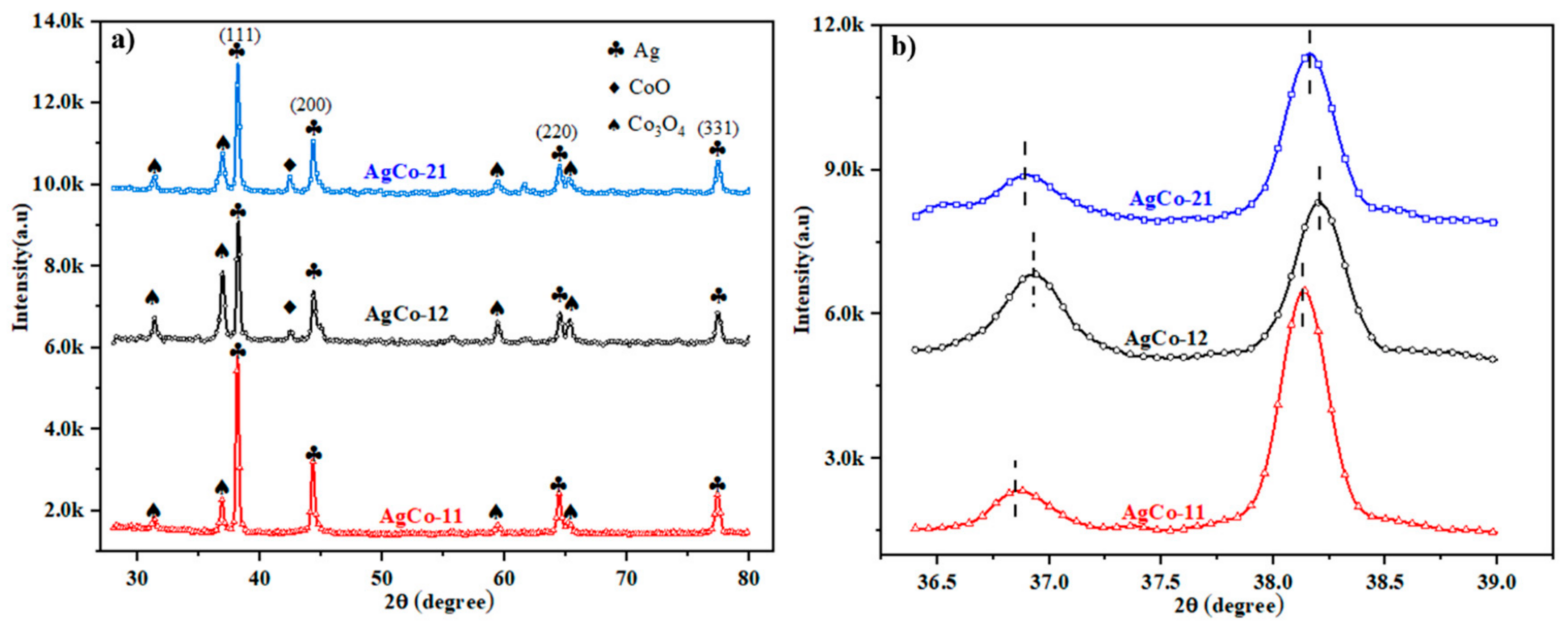
(**a**) XRD pattern of bimetallic Ag-Co synthesized using different modes of combustion synthesis; (**b**) selected 2θ region of the XRD pattern.

Figure 1 shows the XRD pattern of the Ag-Co nanoparticles prepared via three different modes of solution combustion synthesis. The phase identification indicates the presence of metallic Ag and oxides of cobalt in +2 and +3 oxidation states. In all three cases, peaks are present at 38.2°, 44.3°, 64.6° and 77.6°, corresponding to Ag (111), Ag (200), Ag (220), and Ag (331), respectively.

The diffraction pattern of cubic Co_3_O_4_ displays four characteristic bands at 31.28°, 36.6°, 59.21°, and 65.32° that were confirmed with PDF#43-1003. In AgCo-12 and AgCo-21, the Co_3_O_4_ phase was partially reduce to CoO through the second phase of combustion, which produces a reducing atmosphere for further reduction. The presence of CoO in AgCo-12 and AgCo-21 presents a weak diffraction pattern at 43.54°. This result seems inconsistent with the thermodynamic analysis discussed earlier, however, it should be noted that thermodynamic calculations were performed assuming adiabatic and inert conditions, whereas actual combustion experiments were conducted in a beaker placed on a hot plate heater and open to atmospheric air. A close look at the peaks shown in Figure 1b indicates a slight shift in 2*θ* of Ag (111) and Co_3_O_4_ (311) planes to higher values. This could be due to either the existence of lattice strain or to the change in chemical composition owing to formation of the solid solution. The doping of one atom to another causes the rearrangement of the atom periodicity and thus changes cell parameters, causing a shift of diffraction peaks. AgCo-12 shows greater displacement, promoting improved combining of Ag and Co atoms to form alloys that act as transition zones leading to bimetal formation [33,34,35].

**Figure 2:**
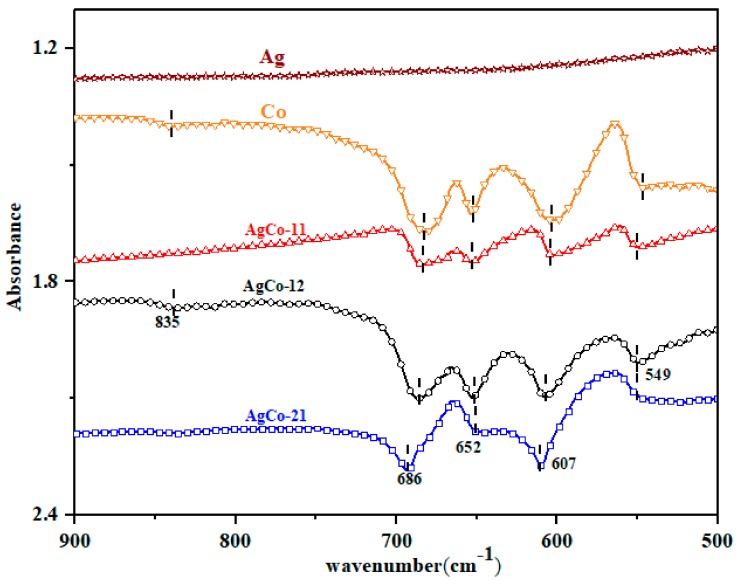
FTIR spectra of the as-prepared bimetals of Ag-Co and their monometals synthesized using different modes of solution combustion synthesis (SCS).

The FTIR spectra in Figure 2 indicate the chemical bonding and functional groups attached to the surface of the as-prepared Ag-Co nanopowders. The spectra of Ag-Co compounds were compared with those of the monometals of combustion-synthesized Ag and Co, prepared with the same fuel ratio. The absorption band between 400 and 655 cm^−1^ corresponds to the metal–oxygen bonds on the catalyst surface. In that case, Ag does not display any absorption peaks, either due to it being in a pure metallic phase or due to the local sintering at the higher fuel ratio weakening the bond vibrations [36,37]. The absorption at 652 and 549 cm^−1^ in cobalt are attributed to the stretching vibration of cobalt–oxygen bonds and confirms the presence of spinel Co_3_O_4_. The vibration spectrum at 652 cm^−1^ is ascribed to tetrahedrally coordinated Co^2+^ (3d^7^), and the 549 cm^−1^ band confirms the octahedrally coordinated Co^3+^ (3d^6^) [38,39]. The presence of unburned carbonyl impurities from the incomplete combustion was affirmed from the weak absorption peak at 835 cm^−1^ [40]. The absorption spectra of monometallic cobalt and AgCo-12 have a close resemblance that indicates that cobalt predominates on the surface of AgCo-12. Moreover, the absorption peaks of AgCo-21 tend to merge into a flat band, confirming the predominant elemental phase of metallic silver on the surface. The existence of peaks indicating spinel Co_3_O_4_ in AgCo-21 is possibly due to a higher molar ratio of cobalt in the overall composition.

**Figure 3:**
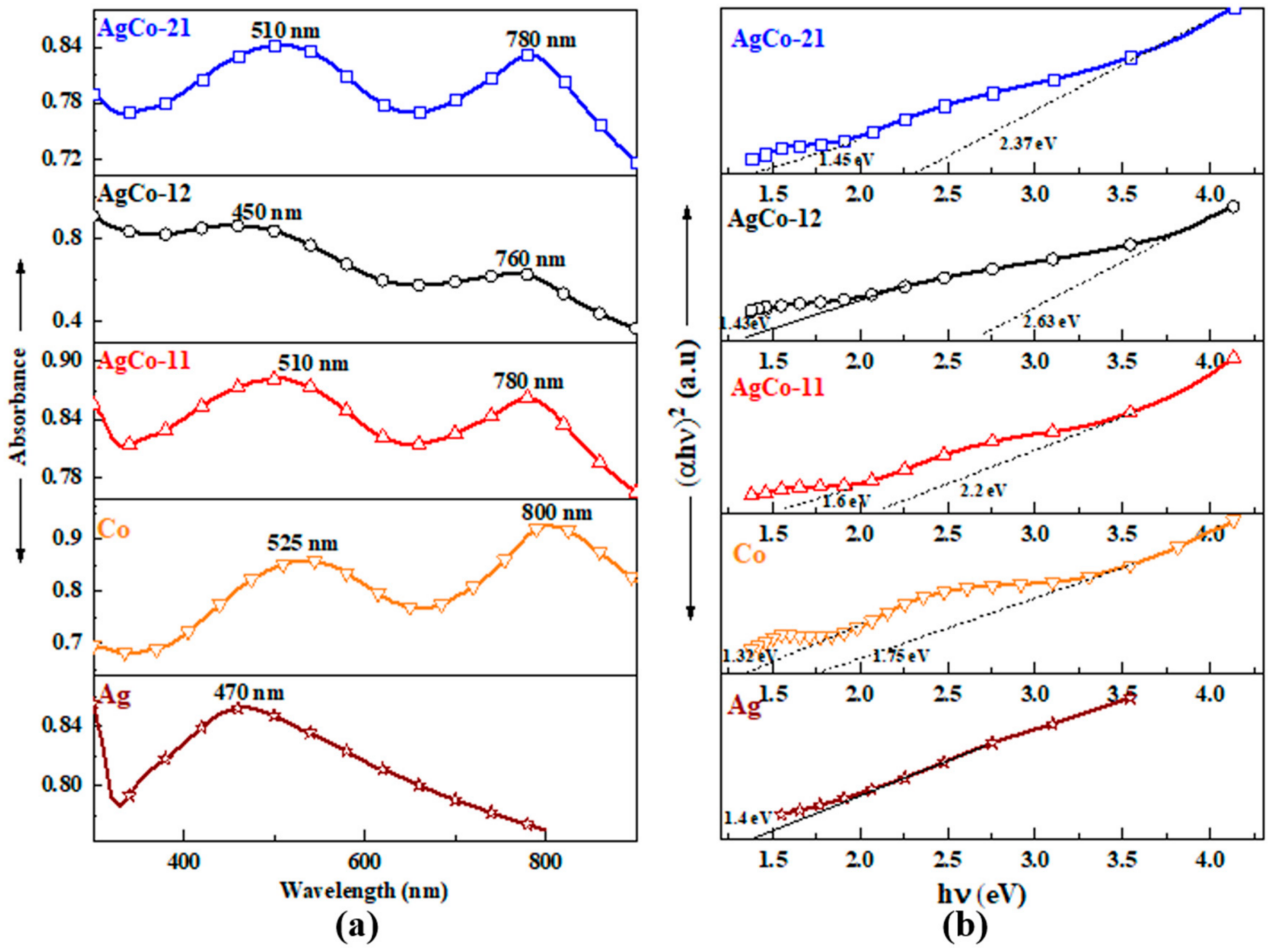
(**a**) UV-Vis absorption spectra of monometals Ag and Co, and alloyed Ag-Co compounds in aqueous solution; and (**b**) their corresponding Tauc plot.

The optical absorbance spectra of all the synthesized catalysts are shown in Figure 3. The surface plasmon resonance spectrum of silver nanoparticles shows a single absorption peak at 470 nm, and that for the cobalt nanoparticles indicates that there are two absorption bands at 540 nm and 800 nm. The lower band at 540 nm can be attributed to the O^−2^ to Co^+2^ charge transfer, whereas the higher band at 800 nm is associated with the O^−2^ to Co^+3^ charge transfer [41,42]. In silver-cobalt systems, an optical absorption band showing only a single surface plasmon resonance peak on the lower band spectrum confirms the alloying between silver and cobalt [43]. The resonance peak of silver at 470 nm and the lower band spectrum of cobalt at 540 nm merge while alloying. The lower band in AgCo-12 shifted to the left (blue shift) when compared to the other two bimetals, which could be due to the quantum confinement of the nanoparticles with a decrease in dimensions [44]. The absorption band gap for all the samples, as shown in Figure 3b, were determined using the Tauc equation [45,46]:

αhν = *A*(hν − E_g_)^n^

where hν is photon energy (eV), in which h is Planck’s constant and ν is vibrational frequency. *A* is the absorption coefficient, E_g_ is the band gap, and n is a constant value, with n = ½ for direct transition and n = 2 for indirect transition. The two band gap values in cobalt are due to the presence of two absorption peaks that point to the interband transition in spinel Co_3_O_4_. The lower band gap corresponds to the transition between O^2−^ and Co^3+^, and higher band is associated with the O^2−^ and Co^3+^ charge transfer [47,48]. The Co^3+^ forms an intermediate band inside the energy gap of spinal Co_3_O_4_. The optical band gap of AgCo-12 was found to be higher when compared to the other compounds, which could be due to the quantum confinement effect with a decrease in the crystallite size of particles.

**Figure 4:**
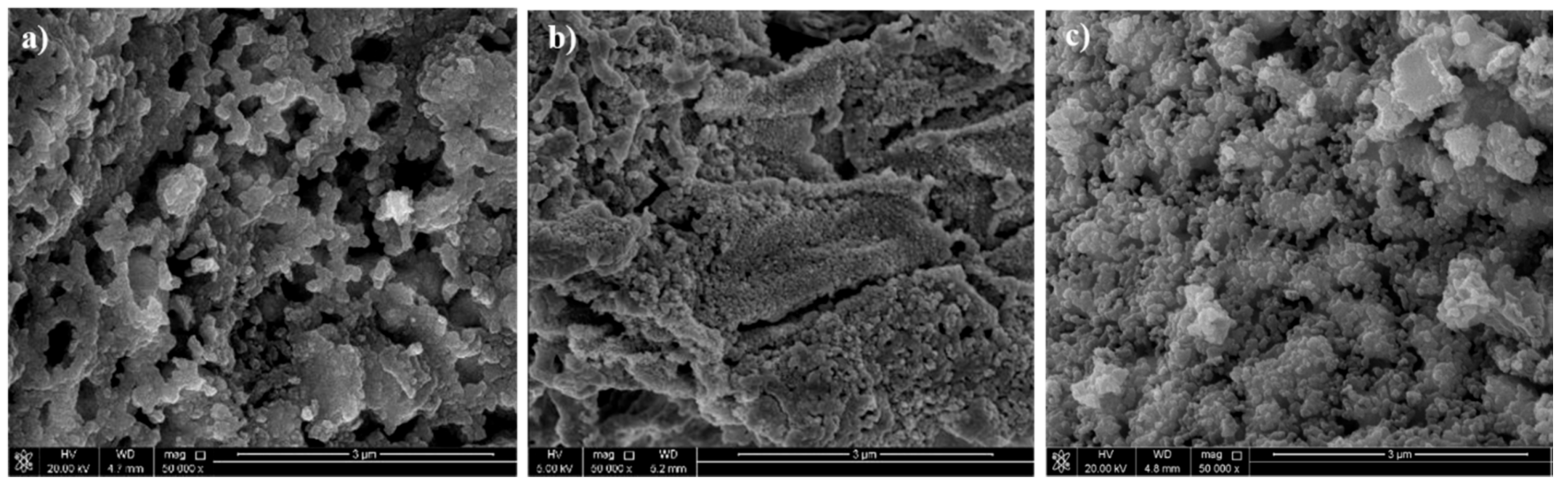
SEM of as-prepared Ag-Co alloys using different modes of SCS. (**a**) AgCo-11; (**b**) AgCo-12; and (**c**) AgCo-21.

SEM micrographs of the Ag-Co alloys synthesized using three different modes of solution combustion synthesis are shown in Figure 4. The synthesis mode plays an important role in tuning the morphology and structure of the nanoparticles. A detailed discussion on the effect of combustion synthesis parameters on the morphology and structure can be found in earlier reports [22,23]. It can be seen that all the nanopowders have a porous network, which is predominantly due to the channels formed by the escaping gases that are released during the combustion process. AgCo-11 shows a broad distribution of smaller particles on larger clusters. AgCo-12 indicates the presence of larger clusters of fine particles. Moreover, AgCo-21 produces a wider distribution of relatively uniformly sized smaller particles that agglomerate to form lumps. The identification of Ag and Co is difficult from visual inspection and requires TEM, along with phase mapping, for a better understanding. The elemental composition of each sample from the EDX analysis is shown in Table S1. It is clear from Table S1 that the composition of Ag and Co in AgCo-11 is approximately the same as the initial composition (1:1.83).

**Figure 5:**
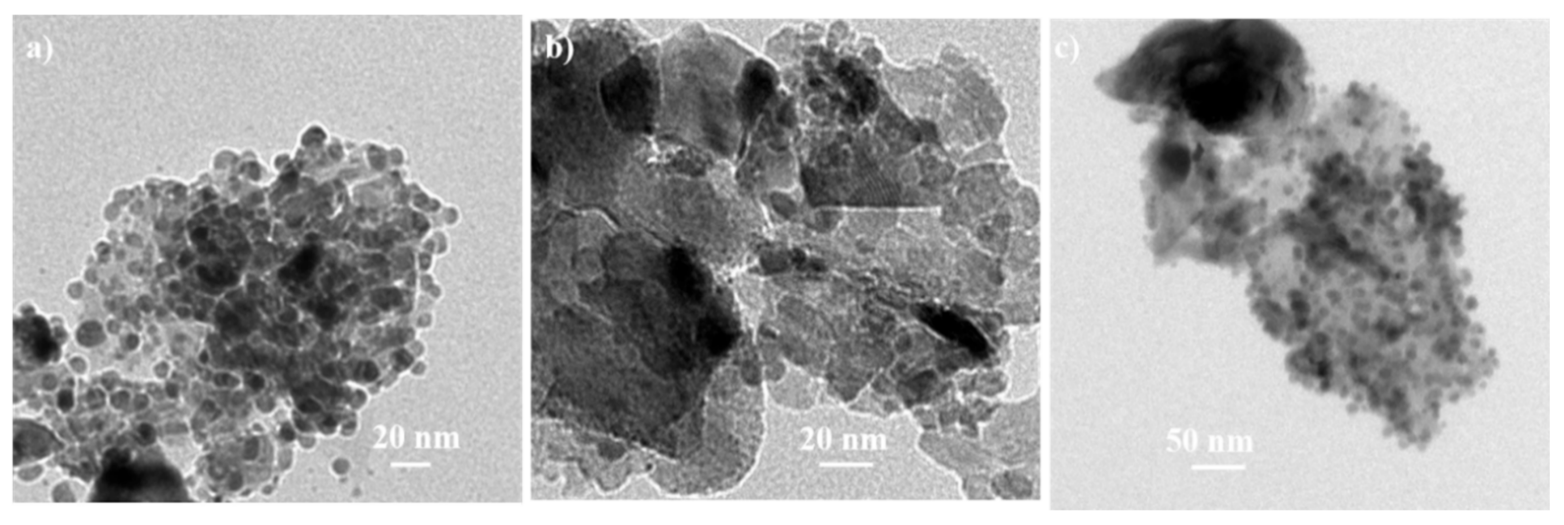
TEM image of synthesized Ag-Co alloys. (**a**) AgCo-11; (**b**) AgCo-12; and (**c**) AgCo-21.

TEM images of as-prepared nanostructures are shown in Figure 5a–c. The phase contrast can be interpreted as the darker particles being silver, while the lighter ones are cobalt, owing to the greater atomic weight of silver (atomic weight = 107.868) compared to cobalt (atomic weight = 58.933). Silver particles are found to be smaller when compared to cobalt, and there appears to be relatively better control of the particle size. Silver particles in AgCo-11 and AgCo-21 are well dispersed over the cobalt particles, as compared to AgCo-21. The agglomeration that is common in combustion synthesis is present to a lesser extent in this Ag-Co alloy; this is due to the step-wise synthesis of these nanoparticles, where the particles synthesized at one stage act as anchors over which the new particles are synthesized in a relatively well-defined way. In AgCo-11, silver particles in the range of 8–15 nm are effectively distributed over cobalt particles, and, for AgCo-12, cobalt particles completely or partially cover the silver particles synthesized in the first wave of combustion. Moreover, in AgCo-21 silver nanoparticles synthesized in the range of 7–14 nm during the second wave combustion are dispersed over the cobalt layer formed initially, as in the case of AgCo-11.

**Figure 6:**
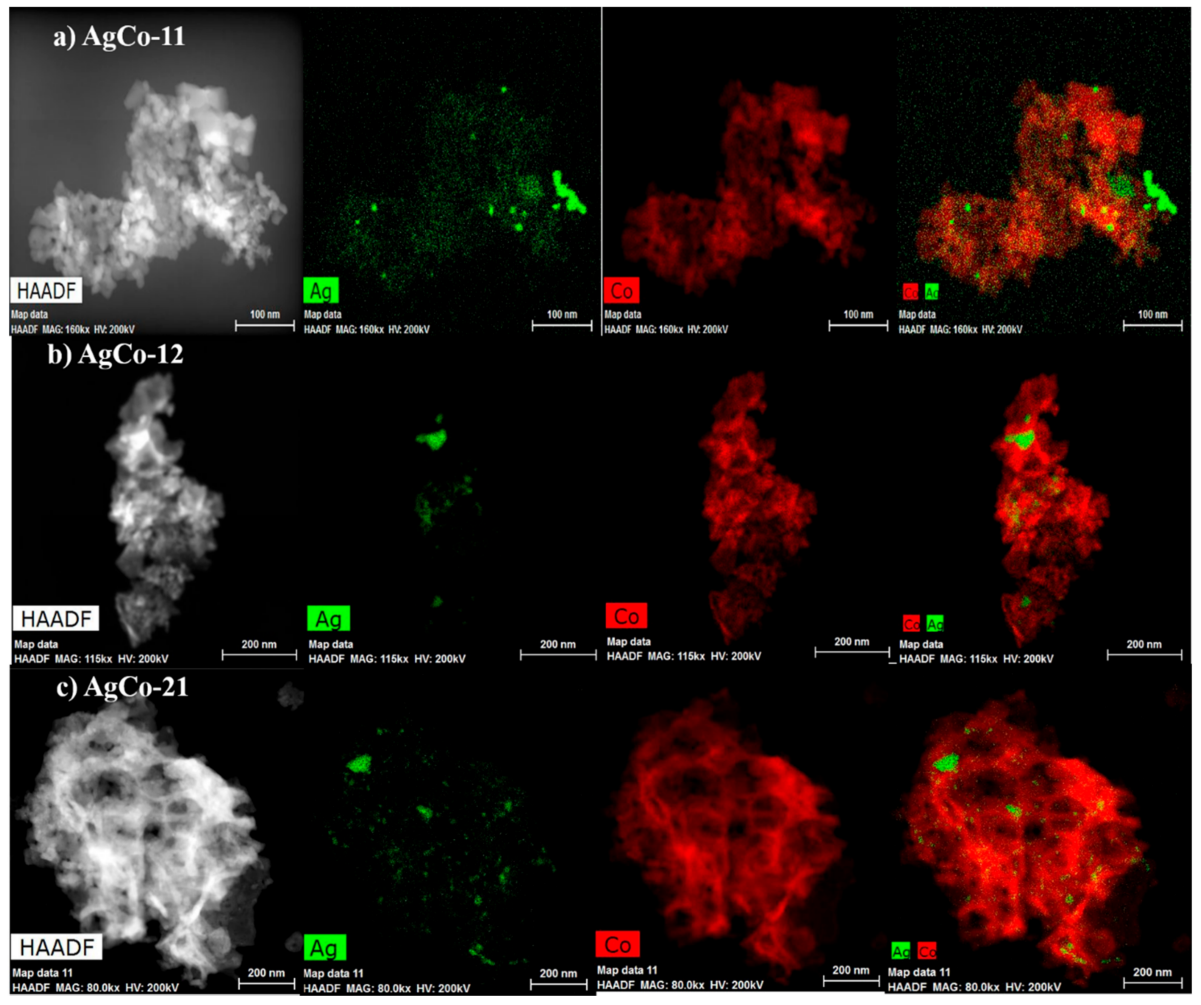
Elemental phase mapping by high-angle annular dark-field scanning transmission electron microscopy (HAADF-STEM) image for the synthesized (**a**) AgCo-11, (**b**) AgCo-12 and (**c**) AgCo-21 nanopowders.

The elemental phase mapping of the synthesized Ag-Co nanoparticles using three different modes of SCS is shown in Figure 6 and gives a better understanding of the alloying and elemental composition. The elemental mapping confirms the presence of Ag and Co throughout the catalyst. The increase in brightness of the STEM image for AgCo-21 could be due to the presence of more silver on the surface of cobalt, which results in a higher-contrast image. As apparent from the TEM image, AgCo-12 has more cobalt particles on the surface, and the size-confined Ag particles are localized below the cobalt surface. It also shows a better controlled alloying for AgCo-12 that is evident from the XRD peak shifting when compared to the other two samples. The surface elemental composition was further evaluated using XPS analysis to confirm the findings from TEM and SEM images.

**Figure 7:**
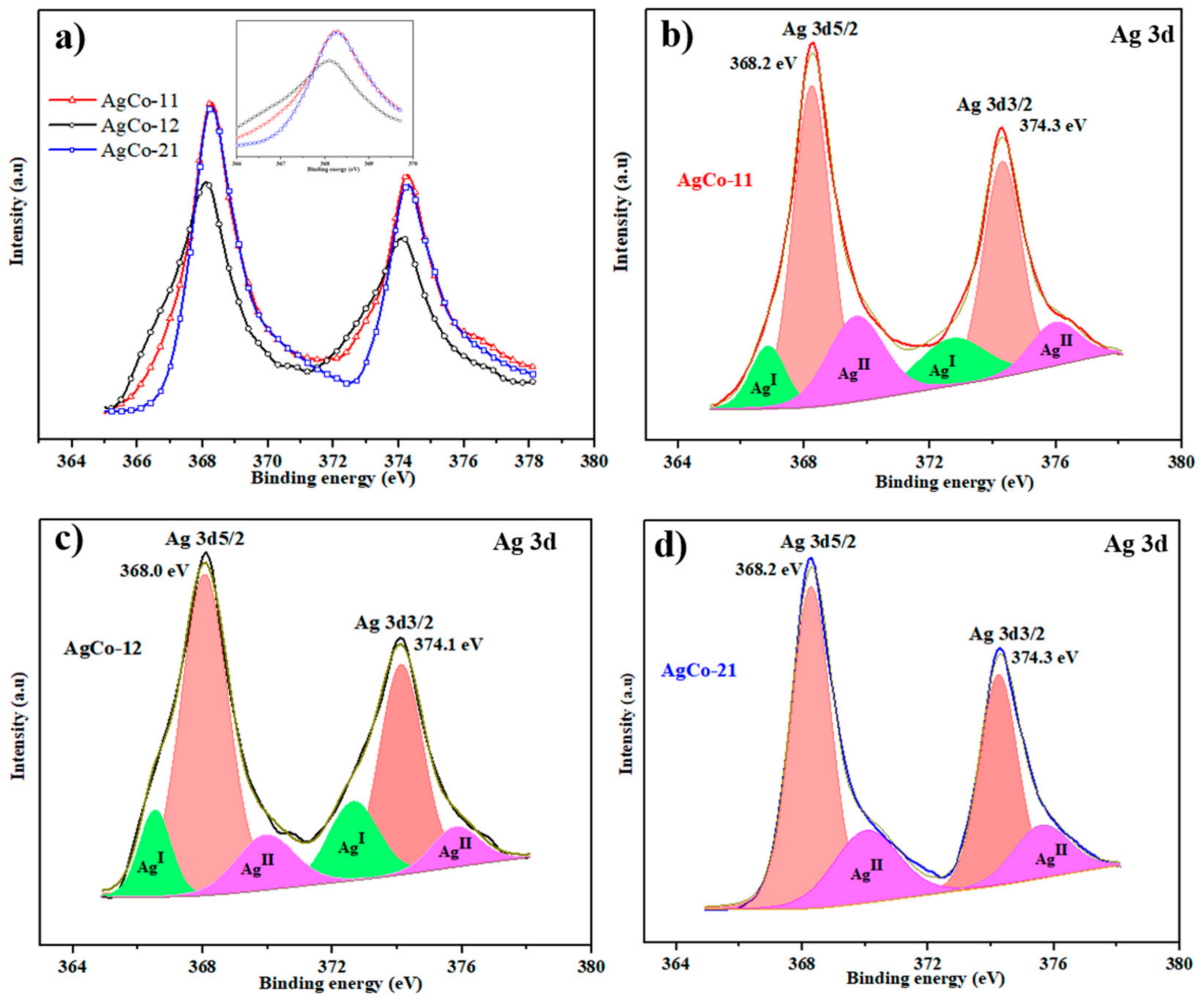
XPS spectrum of Ag 3d in Ag-Co: (**a**) peak intensity spectrum of Ag 3d; (**b**–**d**) deconvoluted spectrum of each Ag 3d.

Figure 7 shows the XPS surface analysis of as-prepared silver-cobalt alloys. The XPS spectrum in Figure 7a shows a maximum intensity of Ag 3d for AgCo-11 and AgCo-21 than AgCo-12. It shows two strong peaks at 368.4 eV and 374.4 eV, with a splitting of 6 eV, that are attributed to the Ag 3d5/2 and Ag 3d3/2 orbitals of metallic Ag [49,50]. The positions of Ag 3d5/2 and Ag 3d3/2 and their corresponding areas are shown in the Table 1. It should be noted that the areas of the Ag 3d peaks for the three Ag-Co samples have a dramatic distinction. The peak area is a function of the number of Ag atoms on the surface. Highly dispersed Ag atoms on the surface cause an increase in the intensity of Ag 3d spectrum lines. From this aspect, the amount of Ag 3d in the surface is decreasing in the order of AgCo-21 > AgCo-11 > AgCo-12. This could be due to the synthesized silver in the first stage of combustion being covered with the cobalt synthesized later. This sequence of combustion reduces the surface Ag content in AgCo-12 compared to the other two cases.

Each Ag 3d level can be deconvoluted by splitting the peaks on the basis of the Gaussian function to calculate the amount of the various oxidation states. The detailed quantitative data of each peak are given in Table S2 of the Supplementary Material provided with this manuscript. After deconvolution, two additional peaks are detected at ~366 eV (~372 eV) and ~369 eV (~375 eV), indicating the presence of Ag_2_O (Ag^I^) and AgO (Ag^II^), along with metallic Ag, which is the major phase (Figure 7b–d) [51,52]. The existence of these oxidized forms can also be correlated with the cobalt on the scanning surface. The ratio of Ag^I^ to Ag^II^ is higher in AgCo-12 than in AgCo-11, which could be due to the better adhesion of oxygen from Co_3_O_4_ on the surface of Ag. The absence of Ag^I^ in the AgCo-21 sample may be due to the presence of more Ag on the surface, and that hinders the oxygen coupling from the cobalt species that are situated beneath it.

**Figure 8:**
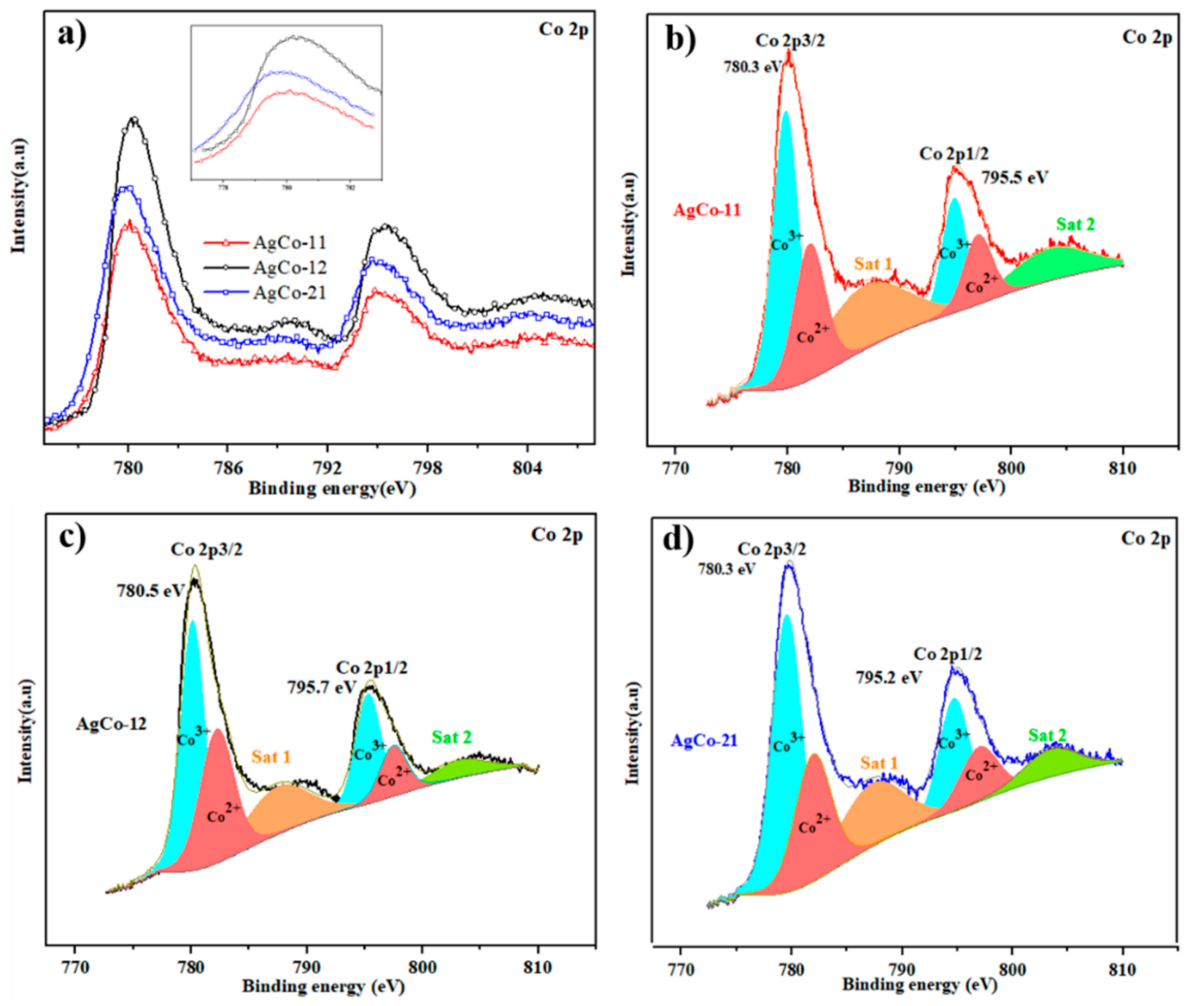
XPS spectrum of Co 2p from bimetallic Ag-Co: (**a**) peak intensity spectrum of Ag 2p and (**b**–**d**) deconvoluted spectrum of each Ag 3d.

It is clear from the Co 2p spectrum (Figure 8a) that the Co concentration on the surface increases in the order of AgCo-11 < AgCo-21 <AgCo-12. The highest content of Co is on the surface of AgCo-12, and this is from the second wave of combustion when cobalt is synthesized on the surface of the previously formed silver. The inset of Ag 3d (Figure 8a) in AgCo-12 has shifted to a lower binding energy, and Co 2p in AgCo-12 has shifted to higher energy value relative to AgCo-11 and AgCo-21. This shift indicates that an electron was transferred from Co to Ag in the alloys, and there exists a strong electronic interaction between the metals in AgCo-12 [53]. The Co 2p curve fitting (Figure 8b–d) confirms the existence of the two spin orbital doublets (Co^3+^ and Co^2+^) and two shake-up satellite peaks. The Co 2p spectrum in Figure 8b–d has two distinct sharp peaks at 780.2 ± 0.6 and 795.6 ± 0.6 eV that correspond to Co 2p3/2 and Co 2 p1/2 with a spin-orbital splitting of ~15.4 eV [54]. The deconvolution of each Co 2p peak indicates the coexistence of Co^3+^ and Co^2+^ at lower and higher binding energies. The area of each deconvoluted peak and the corresponding binding energies are shown in Table S3. The peak area of AgCo-12 has a higher value, and the peak in AgCo-11 has the lowest. The surface ratio Co^2+^/Co^3+^ of AgCo-12 (0.81) was similar to that of AgCo-21 (0.76), but much higher than that of AgCo-11 (0.54). Some of the Co_3_O_4_ formed initially is reduced to CoO (Co^3+^ to Co^2+^) through the second wave combustion, as evident from XRD and thermodynamic calculations, and this is the reason for the trend in Co^2+^/Co^3^ composition on the surface [55]. The same reasoning can be extended to the change in area of shake-up satellite peaks at 789.5 eV and 804.5 eV. These shake-up satellite peaks are characteristic of Co_3_O_4_, and the peak being higher for AgCo-11 and smallest for AgCo-21 could be the reason for the absence of the reduction reaction Co_3_O_4_ to CoO in the former case, and there is a chance that more reduction occurred in the latter case through the second wave of combustion.

**Figure 9:**
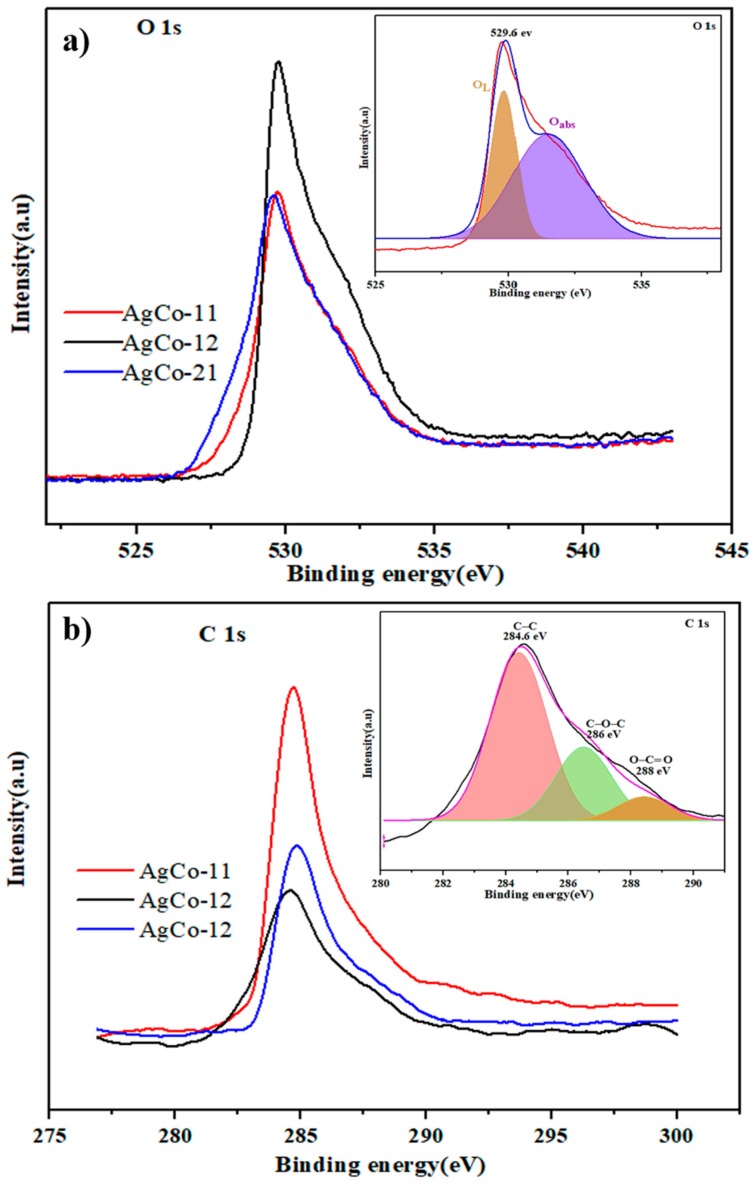
XPS spectrum of (**a**) O 1s (**b**) C 1s of three samples of Ag-Co. Inset shows the corresponding deconvoluted spectrum.

The XPS of the O 1s spectrum in the inset of Figure 9a shows a characteristic peak at 529.6 eV (O_L_) corresponding to the oxygen of the metal crystal lattice (M–O bond), and a noticeable shoulder peak at higher binding energy suggests the presence of absorbed oxygen on the surface [56,57]. The quantitative analysis of O 1s (Figure 9a) in the samples from the three modes reveals maximum intensity of oxygen for AgCo-12, whereas AgCo-11 and AgCo-21 hold the same amount of oxygen. The existence of more O 1s content in AgCo-12 could be from the oxygen bounded to the cobalt (Co_3_O_4_), where AgCo-12 carries more cobalt on the surface, as discussed above.

The deconvoluted XPS peak of the C 1s core-level of sample is illustrated in the inset of Figure 9b. The sharp peak at 284.6 eV indicates the presence of an sp^3^ C–C bond, and smaller shoulder peaks at ~286 eV and ~288 eV show bonding configurations of C–O–C and O–C=O, respectively. The combined spectrum of all the Ag-Co samples indicates the existence of a higher atomic configuration of carbon in AgCo-11, decreasing in the order of AgCo-11 > AgCo-21 > AgCo-12. The decrease in carbon content on AgCo-12 (16.93%) and AgCo-21 (17.93%) when compared to AgCo-11 (29.98%) is due to the combustion of extra carbon in the second wave of synthesis in the SWCS mode, thus reducing the overall C 1s composition. During the second wave of combustion, there is a subsequent rearrangement of elements on the surface, and the higher content of carbon in AgCo-11 causes the reduction of overall metallic/oxide content of Ag and Co on the surface. This is more prominent evidence for the presence of an approximately equal proportion of silver on AgCo-11 and AgCo-21, whereas AgCo-21 has a greater atomic concentration of cobalt on the surface than the AgCo-11 sample. To summarize, it is clear from the results and discussion that the synthesis sequence plays a great role in the surface composition and final structure of the nano-compounds. Moreover, through SWCS, there is better control in alloying and a reduction in the amount of carbon content, which is relatively high in conventional solution combustion synthesis. The nanoparticles synthesized using this modified technique can be used in various catalytic applications, owing to the upgraded surface science and better control of the structure.

## 5. Conclusions

Silver-cobalt (AgCo-11, AgCo-12, and AgCo-21) was prepared using three different modes of solution combustion synthesis with a fixed fuel ratio (*φ* = 1.75). The surface analysis and morphology show the influence of the three different modes on the final structure of the nanoparticles. Based on the preceding discussions, it is clear that the sequence of combustion has a great role in the chemical composition and physical structural characteristics of synthesized nanopowders. XRD analyses indicate that doping one element over other changes the atomic periodicity, and thus changes cell parameters, causing the shifting of diffraction peaks. The vibrational spectra of AgCo-12 and Co_3_O_4_ have similar trends in bonding configurations, indicating the presence of Co_3_O_4_ as the dominant species in AgCo-12. The existence of cobalt in a spinel structure is confirmed from the two optical absorbance peaks at 525 nm and 800 nm. The merging of the silver peak and cobalt spectrum in the lower band indicate the alloying of silver and cobalt in all three modes of synthesis. SEM and TEM confirm the better control of silver particles, with silver content on the surface of AgCo-21 and cobalt on AgCo-12. The phase mapping of AgCo-12 indicates better control of alloying, and this is further verified by XPS analysis through the shifting of the Ag 3d and Co 2p peaks. This peak shifting provides evidence for the strong electronic interaction between Ag and Co, which causes the improved mixing of the metals. Surface analysis of carbon content indicates that the second wave of combustion leads to lower carbon content in the nanopowders, as is clear from the low amounts of carbon in the AgCo-12 and AgCo-21 samples.

## Figures and Tables

**Table 1:** Measured XPS spectrum for Ag 3d and the quantitative analysis.

Alloys	Ag 3d5/2			Ag 3d3/2		
	B.E. (eV)	Area	%Area	B.E. (eV)	Area	%Area
AgCo-11	368.2	4743.26	34.51	374.2	3387	24.64
AgCo-12	368.02	4552.08	39.90	374.0	2974	26.07
AgCo-21	368.27	5488.33	44.73	374.2	3448	28.13

## Supplementary Materials

The following are available online at http://www.mdpi.com/2079-4991/8/8/604/s1.
