# Supplementary material for: Surface Alloying in Silver-Cobalt through a Second Wave Solution Combustion Synthesis Technique
Source: Nanomaterials (Basel). 2018 Aug 9;8(8):604. doi: 10.3390/nano8080604 (PMC6116214; doi:10.3390/nano8080604)
Supplement: Supplementary file 1 [file nanomaterials-08-00604-s001.pdf]

Surface Alloying in Silver-Cobalt through a Second Wave Solution Combustion Synthesis Technique

Anchu Ashok [1], Anand Kumar [2,*] and Faris Tarlochan [1]

[1] Department of Mechanical and Industrial Engineering, College of Engineering, Qatar University, P O Box-2713, Doha, Qatar; anchuashok@qu.edu.qa (A.A.); faris.tarlochan@qu.edu.qa (F.T.)

[2] Department of Chemical Engineering, College of Engineering, Qatar University, P O Box-2713, Doha, Qatar

* Correspondence: akumar@qu.edu.qa; Tel.: +974-4403-4164

Table S1. Elemental composition of catalyst using EDX analysis.

Element	AgCo-11	AgCo-12	AgCo-21
Silver(Ag)	10.41	11.52	40.09
Cobalt(Co)	27.35	44.35	10.73
Oxygen(O)	45.21	38.65	43.29
Carbon(C)	17.03	5.48	5.88

Table S2. Detailed analysis of deconvoluted Ag 3d xps spectrum.

Alloys	Ag^{I*}			Ag^{II*}			Ag^{I**}			Ag^{II**}		
	B.E(eV)	Area	%Area	B.E(eV)	Area	%Area	B.E(eV)	Area	%Area	B.E(eV)	Area	%Area
AgCo-11	366.9	990	7.2	369.54	2206	16.05	372.75	1529.53	11.13	375.96	888.9	6.47
AgCo-12	366.56	933	8.18	369.80	1188	10.42	372.62	1173	10.28	375.8	588.2	5.16
AgCo-21				370.04	3448	28.13				375.60	1294	10.56

Table S3. XPS energy levels of deconvoluted Co 2p and its corresponding areas (in brackets).

Alloys	Co^{3+}	Co^{2+}	Sat 1	Sat 2
**AgCo-11**	779.82 (36003)	781.9 (20882)	786.83 (26925)	803.36 (12444)
	794.89 (13820)	797.01 (9897)		
**AgCo-12**	780.11 (45117)	782.23 (36142)	787.43 (24298)	803.07 (8057)
	795 (22337)	797 (11038)		
**AgCo-21**	779.6 (40795)	782.03 (31004)	787.3 (24151)	803.5 (8842)
	794.7 (19896)	797.01 12222		
